# Metaelectric multiphase transitions in a highly polarizable molecular crystal†
†Electronic supplementary information (ESI) available: Detailed experimental procedures, additional structural information, and additional references. CCDC 1989814–1989818. For ESI and crystallographic data in CIF or other electronic format see DOI: 10.1039/d0sc01687j


**DOI:** 10.1039/d0sc01687j

**Published:** 2020-05-13

**Authors:** Sachio Horiuchi, Shoji Ishibashi, Rie Haruki, Reiji Kumai, Satoshi Inada, Shigenobu Aoyagi

**Affiliations:** a Research Institute for Advanced Electronics and Photonics (RIAEP) , National Institute of Advanced Industrial Science and Technology (AIST) , Tsukuba 305-8565 , Japan; b Research Center for Computational Design of Advanced Functional Materials (CD-FMat) , National Institute of Advanced Industrial Science and Technology (AIST) , Tsukuba 305-8568 , Japan; c Condensed Matter Research Center (CMRC) and Photon Factory , Institute of Materials Structure Science , High Energy Accelerator Research Organization (KEK) , Tsukuba 305-0801 , Japan; d Research & Development Center , Ouchi Shinko Chemical Industrial Co., Ltd. , Sukagawa 962-0806 , Japan

## Abstract

Metaelectric transition, *i.e.* an abrupt increase in polarization with an electric field is just a phase change phenomenon in dielectrics and attracts increasing interest for practical applications such as electrical energy storage and highly deformable transducers. Here we demonstrate that both field-induced metaelectric transitions and temperature-induced phase transitions occur successively on a crystal of highly polarizable bis-(1*H*-benzimidazol-2-yl)-methane (BI2C) molecules. In each molecule, two switchable polar subunits are covalently linked with each other. By changing the NH hydrogen location, the low- and high-dipole states of each molecule can be interconverted, turning on and off the polarization of hydrogen-bonded molecular ribbons. In the low-temperature phase III, the tetragonal crystal lattice comprises orthogonally crossed arrays of polar ribbons made up of a ladder-like hydrogen-bond network of fully polarized molecules. The single-step metaelectric transition from this phase III corresponds to the forced alignment of antiparallel dipoles typical of antiferroelectrics. By the transition to the intermediate-temperature phase II, the polarity is turned off for half of the ribbons so that the nonpolar and polar ribbons are orthogonal to each other. Considering also the ferroelastic-like crystal twinning, the doubled steps of metaelectric transitions observed in the phase II can be explained by the additional switching at different critical fields, by which the nonpolar ribbons undergo “metadielectric” molecular transformation restoring the strong polarization. This mechanism inevitably brings about exotic phase change phenomena transforming the multi-domain state of a homogeneous phase into an inhomogeneous (phase mixture) state.

## Abstract 2

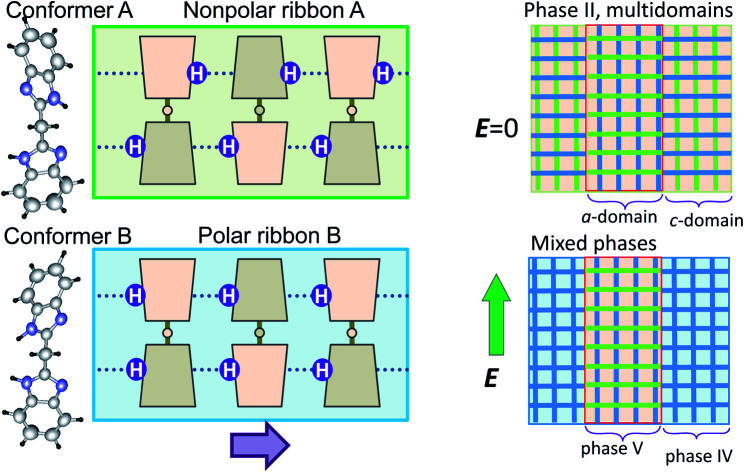
An applied electric field and/or temperature changes can interconvert the low- and high-dipole states of molecules in a metaelectric organic crystal.

## Introduction

Metamagnetic transitions are phenomena exhibiting abrupt increases in magnetization with a magnetic field and observed in itinerant magnets such as Sr_3_Ru_2_O_7_,^1^ as well as in antiferromagnets exhibiting a field-induced spin flop.^2^ Their electric counterpart is just the “metaelectric” transition, which shows a sudden jump in the polarization. Although this terminology has not so explicitly used, it apparently includes the electric-field-induced phase switching phenomena in antiferroelectrics between the antipolar phase and its modified polar one, which are closely comparable in free energy. Such field-induced metaelectric transitions typically manifest themselves by double hysteresis loops in the electric polarization (*P*) *versus* electric field (*E*) curve.^3^ Besides there are more specific examples: such as a ferroelectric with Jahn–Teller structural distortions,^4^ ferroelectric relaxors,^5^ and magnetic-field induced metaelectric transition in a multiferroic material.^6^–^8^

Compared with ferroelectrics of about a century's history, antiferroelectrics have been much less-developed over the shorter period (65 years since the first discovery^9^,^10^ of PbZrO_3_). One of the reasons is their overlooked advantages until their metaelectric transitions are reconsidered recently as promising principles for their applications such as in electrical energy storage,^11^–^13^ large displacement transducers,^14^ and solid state cooling devices.^15^ Likewise for organic systems, an increasing number of publications have reported on new ferroelectric materials^16^–^24^ with expectation for realizing light-weight, flexible, large-area and environmentally benign devices for diverse technological applications such as memories, capacitors, sensors, actuators, and so on. Antiferroelectric characteristics were found in several organic crystals, which have been often regarded as a sort of accidental “byproducts” in search for ferroelectrics.^25^–^27^ Such a perspective has been inverted by our recent discovery of metaelectric transition of squaric acid (SQA),^28^,^29^ which exhibited just the advantages needed for high-efficiency energy storage devices: strongly induced polarization, high switching field, and quite slim hysteresis. Another intriguing antiferroelectric is trifluoromethyl-naphthimidazole exhibiting very large electrostriction.^30^

**Chart 1:**
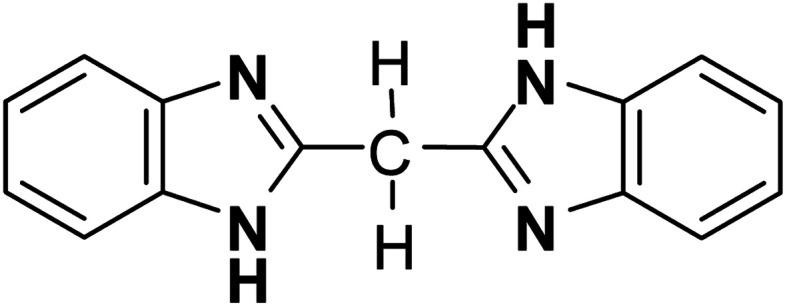
BI2C.

Here we demonstrate a different chemical approach to metaelectric transitions using the so-called “metadielectric” molecule that can adjust its own dipole moments stepwise in magnitudes under the action of external stimuli. For instance, two or more subunits with switchable dipoles can make their total polarization adjustable depending on the relative dipole orientations. The bis-(1*H*-benzimidazol-2-yl)methane (BI2C, Chart 1)^31^,^32^ is one of the bridged bis(benzimidazole)s^33^ carrying two covalently linked imidazole rings. The crystal structure comprises orthogonally crossed arrays of polar ribbons made up of a ladder-like hydrogen-bond network of fully polarized molecules in the lowest temperature phase. Structural analysis reveals that a temperature change can switch on and off the polarization of the hydrogen-bonded molecular ribbons as desired. We also find single and dual metaelectric transitions in the low- and intermediate-temperature phases, respectively. The following two distinct processes are found to be involved in the field- and temperature-induced structural changes. One is the conventional “antiferroelectric” switching with forced alignment of antiparallel dipoles, and the other is the “metadielectric” molecular transformation turning on and off the polarization of each hydrogen-bonded molecular ribbon. We also show that the coexistence of these processes inevitably causes intriguing phenomena that the metaelectric multiphase transitions are reflected by the phase change interchanging the multi-domain state of a single phase and inhomogeneously mixed state of binary phases.

## Experimental

### General

BI2C was prepared by following a reported procedure^31^ and purified by recrystallization from ethanol solution and by vacuum sublimation under temperature gradient. Slow evaporation from the methanol solution gave solvated BI2C·½MeOH (pale yellow needles) crystals, which gradually became opaque by losing MeOH molecules in air. The solvent-free form, which was obtained as the minor product from MeOH, exclusively crystallized as colorless bipyramids from ethanol or isopropanol solution. Note that the use of 99.5% EtOD (ISOTEC Corp.) as the solvent afforded twinning-free large crystals (a few millimeter size) of deuterated form (called BI2C-*d*_2_ hereafter). The degree of deuteration was evaluated by recording the infrared vibrational spectra on a KBr disk using a JASCO FT/IR-6600; the amount of residual undeuterated species was estimated from diminishing band intensity at 1050 cm^–1^ by using the band at 739 cm^–1^ as a relative standard. The alternative method of growing the solvent-free form was vacuum sublimation with a source temperature of 513 K, which is about 90 K higher than that for monomeric 2-methylbenzimidazole. Here the crystals from sublimation are distinguished as the BI2C(s) specimen from recrystallized solvent-free BI2C crystals denoted as BI2C(r/solvent). See Fig. S1 in the ESI† for photographs of the crystals.

The high-sensitive thermal analysis was performed using a differential scanning calorimeter (DSC7000X; Hitachi High-Technologies Corp., Tokyo). The sample was encapsulated in an aluminum pan and heated at a rate of 5 K min^–1^. The temperature was calibrated by using the melting point of indium (429.8 K). The thermograms are corrected by subtracting the blank contributions, which were separately measured on an empty pan under the same experimental conditions.

Prior to the estimation of field-induced electric polarization, a polar periodic lattice has been constructed as the constituent of target ferroelectric structures (degree of polar distortion *λ* = 1) by leaving only the ribbon B having +*x* polarity from the atomic coordinates from the experimentally determined crystal structure. The electronic structure calculations include the energetic relaxation of hydrogen atoms' locations, which are adjusted to the rational bond geometry (N–H, 1.09–1.10 Å) on the hydrogen bonds. The reference paraelectric structures (*λ* = 0) were constructed from averaged molecular structures for *λ* = ±1. Electronic polarization was evaluated using the Berry phase approach^34^,^35^ with the QMAS code^36^ based on the projector augmented-wave method^37^ and the plane-wave basis set. To describe the electronic exchange–correlation energy, the Perdew–Burke–Ernzerhof (PBE) version of the generalized gradient approximation (GGA)^38^ was used. The total polarization is obtained as the sum of electronic polarization and ionic polarization.

### Electric measurements

All electric measurements were performed on as-grown single crystals with painted gold or silver electrodes. The most developed crystal faces are parallel to the (101)_t_ or one of its equivalent planes (the Miller indices in the tetragonal setting are distinguished from those in the monoclinic setting henceforth by adding the subscript “t”). Prior to the measurement with *E*‖(100)_t_ configuration, the top and bottom edges of the bipyramid were easily cleaved with a blade along the (001)_t_ plane, which is parallel to the bottom plane of the pyramid. The resultant plates were further cleaved along the (100)_t_ plane at both ends for flat electrodes. Note that these procedures are entirely the same as those of the (pseudo)tetragonal SQA crystals^28^ owing to a very similar crystal growth habit.

The dielectric permittivity was measured with an LCR meter (Precision E4980A, Agilent Corp.). The electric polarization–electric field (*P*–*E*) hysteresis curves were collected on a ferroelectrics evaluation system (FCE-1; Toyo Corp.) consisting of a current/charge–voltage converter (Model 6252), an arbitrary waveform generator (Biomation 2414B), an analog-to-digital converter (WaveBook 516), and a voltage amplifier (HVA4321; NF Corp.). The measurements at room temperature and higher temperatures were performed with a high-voltage triangular wave field and various alternating frequencies. The crystals were immersed in silicone oil to avoid atmospheric discharge at high electric fields (>30 kV cm^–1^).

### Crystallographic studies

Both the collection of the X-ray diffraction data at room temperature and the assignment of the crystallographic axes of the bulk single crystals were performed using graphite-monochromated Mo Kα radiation (*λ* = 0.71073 Å) and a four-circle diffractometer equipped with a two-dimensional detector: a hybrid pixel detector (AFC10 with PILATUS200K; Rigaku Corp.) or a CCD area detector (Mercury 70; Rigaku Corp.). The CrystalStructure crystallographic software packages (Molecular Structure Corp. and Rigaku Corp.) were employed for the direct method and refinement of the structures. The final refinements of the non-hydrogen atoms were performed with anisotropic thermal factors. The hydrogen-bonded hydrogen atoms of BI2C were found with differential Fourier synthesis and calculated in their ideal geometrical positions. The crystallographic data and experimental details are summarized in Table S1.† The ESI also includes the structural analysis of the solvated BI2C·½MeOH crystal (Fig. S2†).

Temperature-dependent structural changes were also examined for the BI2C crystals. High-resolution diffraction data were collected with a Rigaku DSC imaging plate system using Si double-crystal monochromatized synchrotron radiation (*λ* = 1.00 Å) at the BL-8A and BL-8B beamlines of the Photon Factory (PF), High-Energy Accelerator Research Organization (KEK). The monochromatized beam was focused using a bent cylindrical mirror made of a Si crystal coated with Rh to produce a focused beam size of 0.3 mm (vertical) × 0.7 mm (horizontal). The incident beam was collimated to 0.3 mm × 0.3 mm by the slits set just upstream to the sample. The temperature of the crystal attached to a glass fiber was controlled by using a nitrogen gas blower. The full data collection for the structural analysis was performed at different temperatures to compare the structural changes before and after the phase transition. The Rapid-AUTO program (Rigaku Corp.) was employed for the two-dimensional image processing of the synchrotron X-ray data. The CrystalStructure software package was employed for the direct method and refinement of the structures.

## Results and discussion

### Thermal analysis

**Fig. 1:**
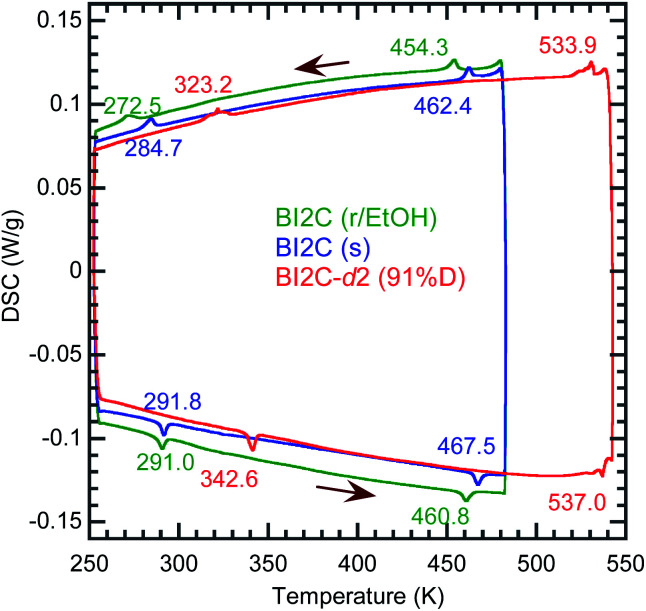
Differential scanning calorimetry (DSC) thermographs of BI2C: recrystallized (r/EtOH), sublimed (s), and deuterated (91% D) crystalline solids. Arrows indicate the directions of temperature changes at a rate of 5 K min^–1^.

The temperature-induced phase transitions were identified by using the differential scanning calorimetry (DSC) thermograms of polycrystalline samples (Fig. 1); each of the recrystallized BI2C(r/EtOH) and sublimed BI2C(s) specimens exhibit two couples of exothermic and endothermic peaks at high temperature (*T*_I/II_ ∼ 458 and 465 K) and just below room temperature (*T*_II/III_ ∼ 282 and 288 K, respectively). Therefore, three crystal phases below the decomposition point (∼582 K) are designated as I, II, and III from the high-temperature side. The temperature hysteresis is indicative of a first-order phase transition. Calculations of the peak area gave a very tiny latent heat of 84 ± 2 J mol^–1^ and 87 ± 2 J mol^–1^ at *T*_I/II_ and *T*_II/III_, respectively. The BI2C(r/EtOH) and BI2C(s) specimens are regarded as identical in polymorphism considering their quite similar polarization and thermal properties. The latter seems to be better in the crystal purity and/or quality judging from the slightly higher transition points with sharper exothermic and endothermic peaks. The phase transition temperatures of BI2C(r/solvent) specimens somewhat depended on the solvent alcohols used, and their best ones employed in Fig. 1 were found on the specimen BI2C(r/EtOH) grown in ethanol. These observations could be explained by the strong sensitivity of phase transition temperature to the stress or pressure considering the tiny latent heat despite the significant temperature hysteresis.

According to the DSC thermogram (Fig. 1), the substitution of all the NH hydrogen atoms for deuterium significantly increases both the phase transition temperatures *T*_I/II_ and *T*_II/III_. The strong deuteration effects suggest that some proton transfer processes are involved in both phase transitions. Compared with *T*_II/III_ = 288 K and *T*_I/II_ = 465 K of the BI2C(s) specimen (based on the midpoints of thermal hysteresis, Fig. 1 and S3†), the phase transition temperatures (their increments) of BI2C-*d*_2_ are *T*_II/III_ (Δ*T*_II/III_) = 313 K (+25 K) and 333 K (+45 K); *T*_I/II_ (Δ*T*_I/II_) = 504 K (+39 K) and 535 K (+70 K) for the 67% D and 91% D specimens, respectively. Consequently, the phase II at room temperature is replaced by the phase III with deuteration. The I/II phase transition revealing a much stronger deuteration effect is reminiscent of the isotope effects on the Curie temperatures for many hydrogen-bonded (anti)ferroelectrics.^39^–^42^ Then, the phase I is likely related to the proton-disordered paraelectric state.

### Reassessment of the phase II structure

The crystal structure of BI2C reported by Duan *et al.*^32^ (CSD ref. codes: XAVFEG, XAVFEG02) has a body-centered tetragonal lattice and the space group *I*4_1_/*a* (#88) symmetry at room temperature (phase II). In contrast, our re-examinations of X-ray structural analysis revealed that the genuine crystal symmetry is reduced to the monoclinic, space group *P*2_1_/*c* (#14) (two-rank subgroup of *I*4_1_/*a*). See the ESI (Table S1†) for detailed structural refinement data on both the recrystallized (BI2C(r/EtOH)) and sublimed (BI2C(s)) specimens. For both specimens, the broken systematic absences of *hkl*: *h* + *k* + *l* = 2*n* + 1 indicated that the crystal lattice is of a primitive type instead of a body-centered one. The phase II (the point group, 2/*m*) is regarded as potentially ferroelastic, whose prototype point group is 4/*m*. While this symmetry reduction is accompanied by the deviation of the angle *β* from the right angle to 90.531(1)° {90.610(3)°}, the unit cell parameters *a* and *c* show a small difference (*a*/*c* = 1.00057(7) {1.0002(1)}) for the BI2C(r/EtOH) {BI2C(s)} specimen. Therefore, crystal twinning as a multidomain state was easily overlooked in conventional diffraction experiments. In contrast, all the as-grown crystals examined were found to be twinned by the high-resolution diffraction experiments using synchrotron X-rays (Fig. S4†). The single domain form was obtained by cutting a BI2C(r/EtOH) crystal (grown from methanol solution) into a smaller piece (the crystals are easily cleavable parallelly to the (100), (010), and (001) planes). The BI2C(r/EtOH) single crystal was used also for structural analysis at low temperatures.

**Fig. 2:**
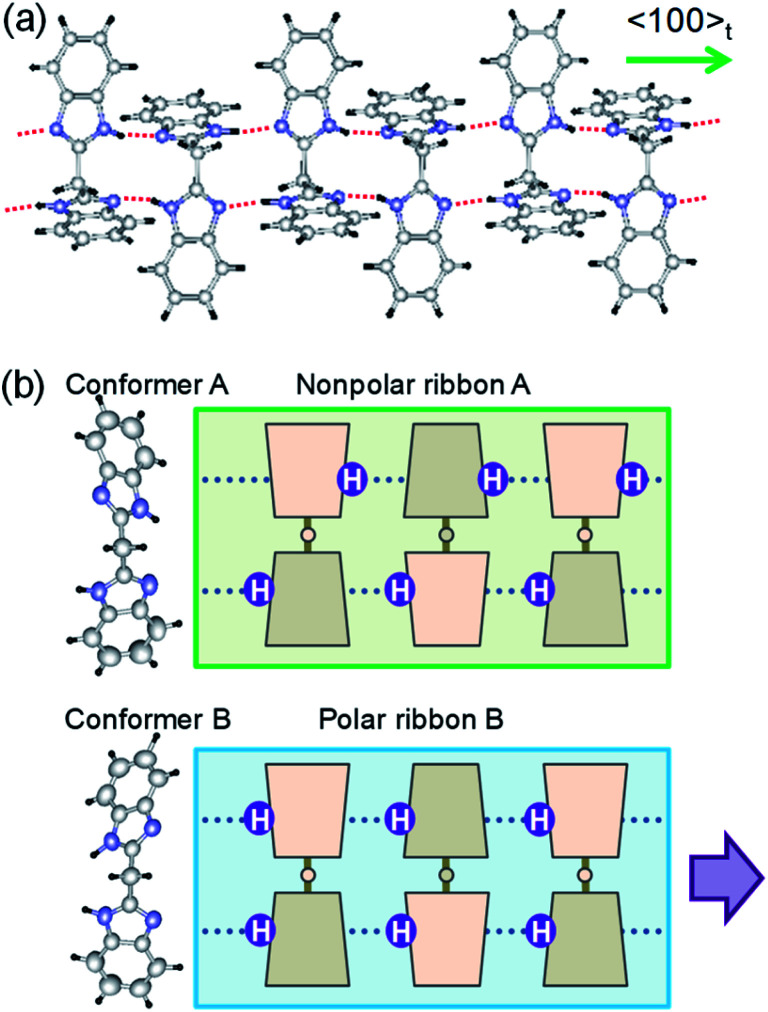
Molecular structures and hydrogen-bonded arrangements in the BI2C crystals. (a) Ribbon-like architectures constructed from intermolecular hydrogen bonds (red dotted lines). (b) Two conformers with different NH hydrogen locations viewed along the 2-fold rotation axis of the bridging C–CH_2_–C tetrahedron (left). Schematic structures of ribbons with and without local polarization (thick arrow).

The monoclinic structure comprises four crystallographically independent molecules (denoted as A_1_, A_2_, B_1_, and B_2_ in Fig. S5†). All the BI2C molecules are similarly bent at the central methylene unit (see Table S2† for the torsion angle). The two benzimidazole units separated by the methylene bridge are individually involved in the intermolecular hydrogen bonds. A ladder-like ribbon is formed by the hydrogen-bonded doublet sequences and the covalent-bonding bridge (Fig. 2a). Compared with the intermolecular N···N distances in the ferroelectric or antiferroelectric benzimidazole crystals (2.80–2.97 Å),^26^ the corresponding distances in the BI2C crystal (2.77–2.86 Å) are found to be comparable or slightly shorter and then would warrant field-induced prototropic switching as well. The ribbons are orthogonally arranged by extending parallel to either the monoclinic *a* or *c* axis (*a*_t_ or *b*_t_ axis in the tetragonal setting) (Fig. S6†).

The crystal comprises molecules of two different conformers (denoted as A and B hereafter) in a 1 : 1 ratio. The conformer A (*i.e.* molecules A_1_ and A_2_) adopts pseudo-*C*_2_ symmetry, in which the orientation of NH hydrogen atoms is opposite between two benzimidazole rings when viewed along the 2-fold rotation axis of the bridging C–CH_2_–C tetrahedron. Due to the antiparallel orientation of NH hydrogen atoms, the net dipole moment of the conformer A should be tiny along each ribbon (Fig. 2b). The conformer B (molecules B_1_ and B_2_) adopts a similar conformation but its pseudo-*C*_s_-symmetry with the aligned orientations of NH hydrogen atoms optimizes its dipole moment. In the actual crystal, the interconversion of one conformer to another is allowed not by the conformational isomerism but rather by the prototropy *via* the hydrogen bonding as shown below. In each molecule, two imidazoyl rings are differently twisted against the bridging C–CH_2_–C, as can be seen from the difference in the corresponding CCCN torsion angles (Table S2†). The rotational structural flexibility around the bridge is reflected in the variations in these twisting angles with molecules as observed.

**Fig. 3:**
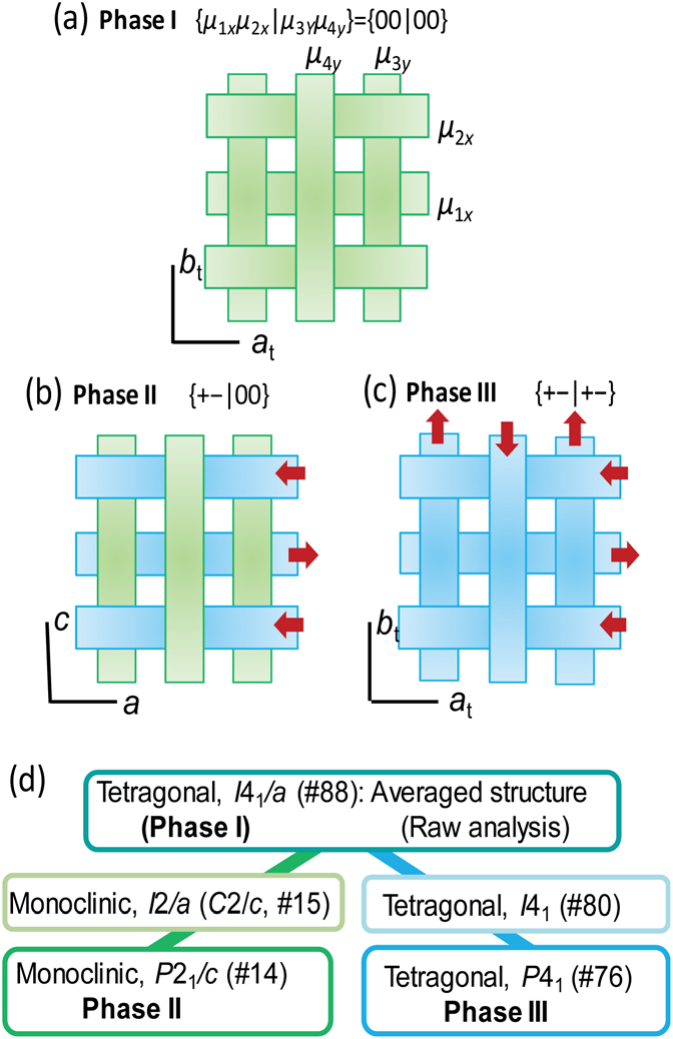
Schematic three-dimensional arrangements of ribbons in (a) a previously reported tetragonal (symmetry-averaged) crystal structure (and possibly phase I), (b) the true monoclinic phase II structure determined by the high-resolution diffraction experiments, and (c) the phase III structure. (d) Group-subgroup interrelationship of space group symmetry between the phase II and III structures.

The conformers A and B are aggregated into separate ribbons (denoted as ribbons A and B, respectively), which are orthogonal to each other (Fig. 3b). The reduction from tetragonal to monoclinic symmetry stems from the inequivalence of ribbons A and B. The ribbons A are exactly nonpolar by symmetry and parallel to the slightly longer *a*-axis. The ribbons B have an aligned NH orientation and then strong polarization along the shorter *c*-axis. As shown by the thick arrows in the figure, the polarities of ribbons B are alternating, constructing an antiferroelectric order. One can see some analogies with the magnetic system of spin *S* = 1, because the ribbons can adopt three different states in terms of their dipole moments *μ*: “0” state for the nonpolar ribbons A and “+” or “–” state for the dipolar ones B. Here we adapt these symbols for the dipole moments *μ*_1*x*_ and *μ*_2*x*_ of two ribbons parallel to the *a*-direction and *μ*_3*y*_ and *μ*_4*y*_ of the rest (see Fig. 3a) to describe the three-dimensional dipole arrangements of four ribbons comprised in the unit cell. For instance, the phase II structure shown in Fig. 3b can be expressed as {*μ*_1*x*_*μ*_2*x*_|*μ*_3*y*_*μ*_4*y*_} = {+–|00}. The nonpolar and strongly polarized states of ribbons should be nearly degenerate in free energy, considering their coexistence in the same crystal lattice. Therefore, electric as well as thermal stimuli are expected to cause the metadielectric molecular switching of BI2C between the conformers A and B. Also note that the tetragonal symmetry employed in preceding studies is the artifact arising from the multidomain states and represents the structure averaged over the two monoclinic crystal structures of the original and its 90°-rotated configurations.

### Phase III structure

In order to get insight into the switching mechanism below, we also scrutinized the temperature-induced structural transformations. Based on the observed isotope effects, the crystal structure of the phase III is ready from the deuterated BI2C-*d*_2_ crystal at room temperature. Besides, variable-temperature diffraction experiments were performed between 160 and 400 K (Fig. S7†) on a twinning-free BI2C(r/EtOH) crystal using a synchrotron X-ray source. Also, the crystal symmetry of phase I was tested at a higher temperature (up to 468 K) using a different crystal specimen.

**Fig. 4:**
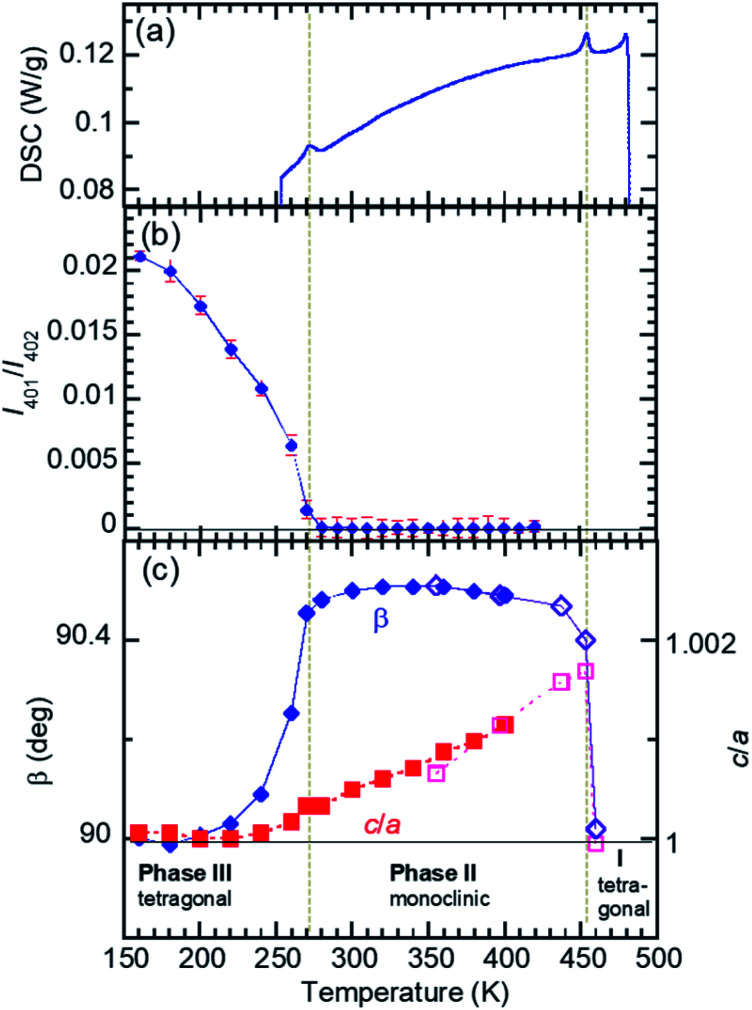
Reduction of lattice symmetry of a recrystallized BI2C(r/EtOH) crystal through successive phase transitions. (a) DSC thermogram (cooling run) indicating the phase transition temperatures (vertical dotted lines). (b) Transformation from a body-centered to a primitive form probed by the integrated intensity of (401) reflection (*h* + *k* + *l* = odd). (c) Deformation from a tetragonal to a monoclinic form demonstrated by the deviation from *β* = 90° and *c*/*a* = 1 (dotted line). The data on two specimens are distinguished by open and filled marks.

Both the transition from phase II to I and that from II to III are accompanied by the restoration of tetragonal lattice symmetry (Fig. 4b). The systematic absences for the phase I structure are compatible with the space group *I*4_1_/*a*, the same as the reported room temperature structure noted above. (Gradual sublimation of the crystal prevented the data collection for full structural analysis of the phase I structure.) On the other hand in the phase III, systematic absences (*hkl*: *h* + *k* + *l* = 2*n* + 1) (Fig. 4a) are broken and then the tetragonal lattice is not body-centered but of a primitive type. The phase III structure at *T* = 200 K is isomorphous to that obtained from the deuterated crystal at room temperature (Fig. 3c) as expected above. Although the observed systematic absences (00*l*: *l* ≠ 4*n*) suggested the candidate noncentrosymmetric space groups of *P*4_1_ (#76) and *P*4_3_ (#78), the component elements, C, H, and N alone do not have an anomalous dispersion effect of X-rays enough to uniquely identify which space group represents the absolute structure. Note that the symmetry lowering upon heating (*i.e.* from tetragonal to monoclinic symmetry) is a rare phenomenon (for instance, see ref. 43). While the space group symmetries of both phases II and III are the two-rank subgroup of *I*4_1_/*a*, they belong to completely different group-subgroup routes from each other (Fig. 3d). This fact justifies the improper ferroelectric and first order nature of the phase transition.

The crossed array of ribbons in the phase III structure is occupied solely by the strongly polarized BI2C molecules of conformer B. The 4-fold screw symmetry cancelled out the strong dipole moment lying along the ribbons with each other, constructing a {*μ*_1*x*_*μ*_2*x*_|*μ*_3*y*_*μ*_4*y*_} = {+–|+–} structure as schematically shown in Fig. 3c. The allowed polarity is only normal to the hydrogen-bonded ribbons (*P*‖*c*_t_), and then the corresponding spontaneous polarization, if present, should be tiny. It is evident that this crystal structure represents a new antiferroelectric phase distinguished from the field-induced ferroelectric one.

**Fig. 5:**
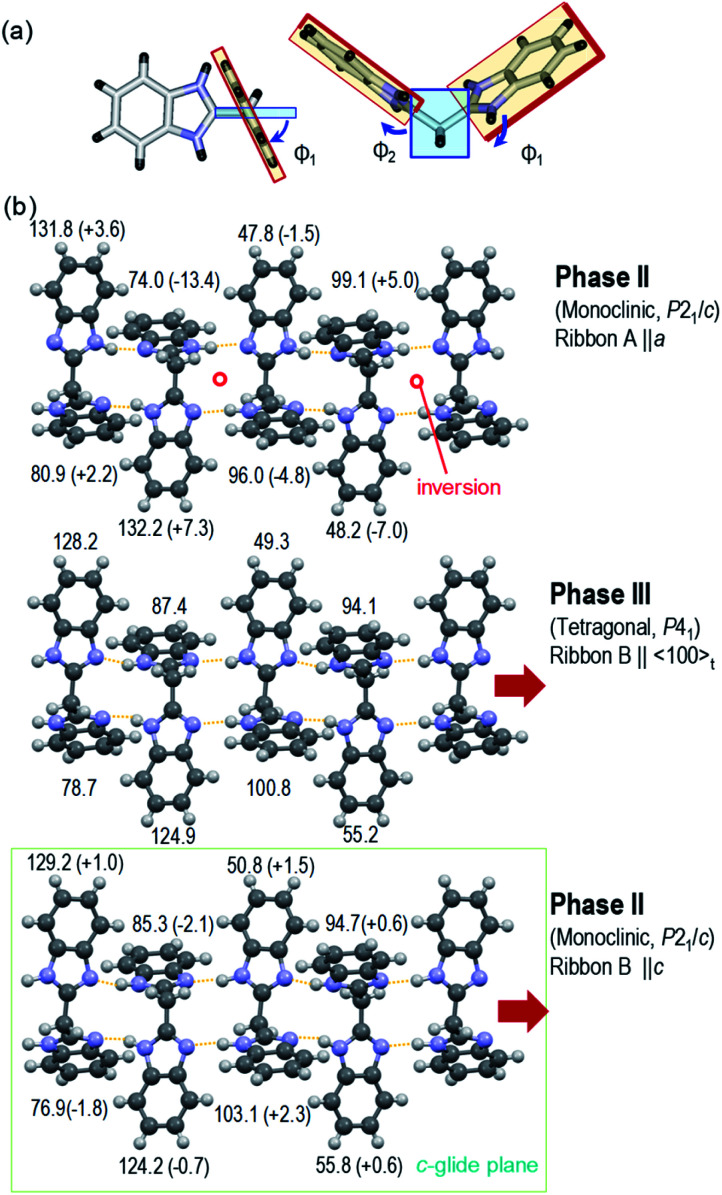
Variations of characteristic dihedral angles *Φ*_*i*_ in the BI2C(s) (phase II) and BI2C-*d*_2_(91% D) (phase III) at room temperature. (a) Definition of *Φ*_*i*_ derived from averaged CCCN torsion angles (see also Table S2†). (b) Comparison of *Φ*_*i*_ between the tetragonal phase III (middle panel, ribbon B) and the monoclinic phase II (top panel, ribbon A; bottom panel, ribbon B). The changes of angle Δ*Φ*_*i*_ from the tetragonal form are given in parentheses.

The crystal structure of deuterated BI2C-*d*_2_ at room temperature was compared with that of BI2C, in order to inspect how the phase II-to-III transformation is accompanied by the local conformational changes of molecules in addition to the protons' configurations. The representative parameters of the conformational flexibility are the CCCN torsion angles of the imidazolyl rings against the methylene bridges, as noted above and in Table S2.† Here, the dihedral angles *Φ*_*i*_ (taken as positive 0–180°) are defined by averaging the torsion angles and given in Fig. 5. Compared with the tetragonal phase III (middle panel of Fig. 5b), the ribbon B extending on the *c*-glide plane retains the same NH configurations as the conformer B and shows negligibly small angular changes Δ*Φ*_*i*_ ∼ –2.1 to +2.3° in the monoclinic phase II (bottom panel). On the other hand, the transformation of the conformer B into A induces the inversion symmetry in the ribbon A and causes much larger conformational changes (Δ*Φ*_*i*_ ∼ –13.4 to +7.3°, top panel).

### Dielectric properties

**Fig. 6:**
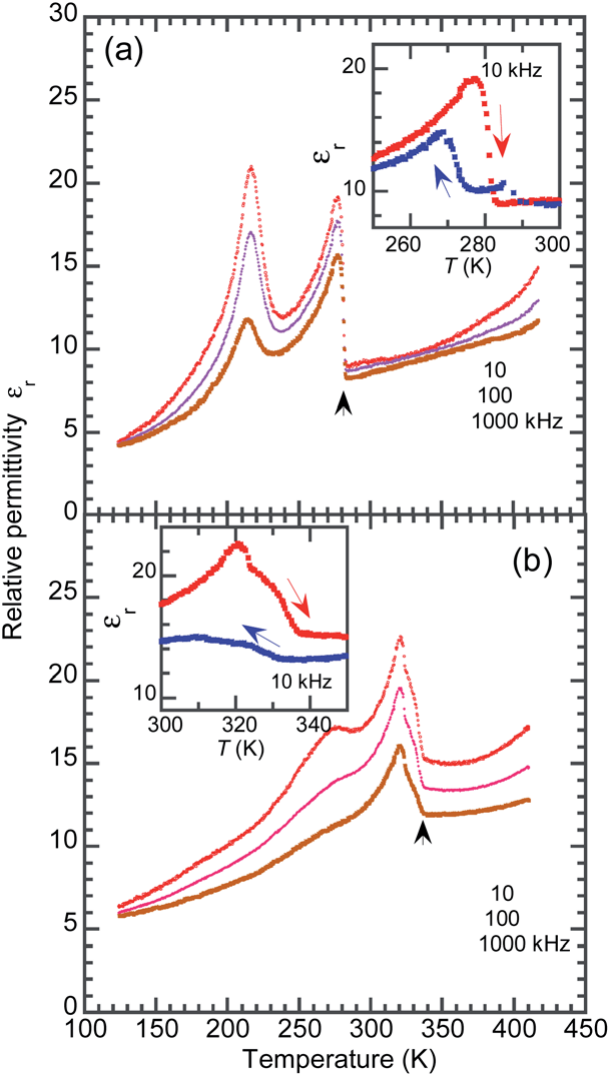
Temperature-dependent relative permittivity of (a) BI2C(r/EtOH) and (b) deuterated BI2C-*d*_2_(91% D) crystals measured with the ac frequency of *f* = 10, 100, and 1000 kHz and *E*‖‖〈100〉100‖〈100〉_t_ configuration (in the heating runs). The inset in each panel indicates the thermal hysteresis of permittivity measured at *f* = 10 kHz between the cooling and heating runs.

The temperature-dependent dielectric permittivity (Fig. 6) was collected with the applied ac electric field configurations of *E*‖‖〈100〉100‖〈100〉_t_. Note that the crystallographic directions are represented in the pseudo-tetragonal lattice setting as notified with the subscript “t” hereafter, considering the twinning which mixes the tetragonal *a*_t_ and *b*_t_ axes. Using the as-grown BI2C specimens, permittivity was measured also with field configurations of *E*‖‖〈101〉101‖〈101〉_t_, which was tilted by 40.7° from the hydrogen-bonded sequence (Fig. S8†). The transitions between the phases II and III are identified by the abrupt jump in the temperature-dependent permittivity. The permittivity jumps exhibit a pronounced thermal hysteresis as shown by the inset in Fig. 6, and are indicative of a first order phase transition again. There are no signatures of the Curie–Weiss behavior characteristic of the proper ferroelectric or antiferroelectric phase transition. Instead, the rounded peak with a frequency dispersion of permittivity signifies the existence of some dielectric relaxations at around 220–230 K. These anomalies are not relevant to a phase transition, because neither the high-sensitive thermal analysis nor the variable-temperature diffraction experiments detected any corresponding thermal events. Because the dielectric relaxation depends on crystal growth conditions (Fig. S8 and S9†), its origin might be extrinsic and affected by some crystal imperfection embedded during the crystal growth and/or structural phase transition near room temperature. The deuteration significantly increases the temperature of the relaxation peak as well as the phase transition temperature (Fig. 6b), whereas it changes little the overall temperature dependence. Regarding the thermal activation of dipolar fluctuation, the monotonous increase of permittivity with temperature in the phase II is consistent with our expectation of the proton-disordered paraelectric state in the phase I.

### Polarization switching properties

**Fig. 7:**
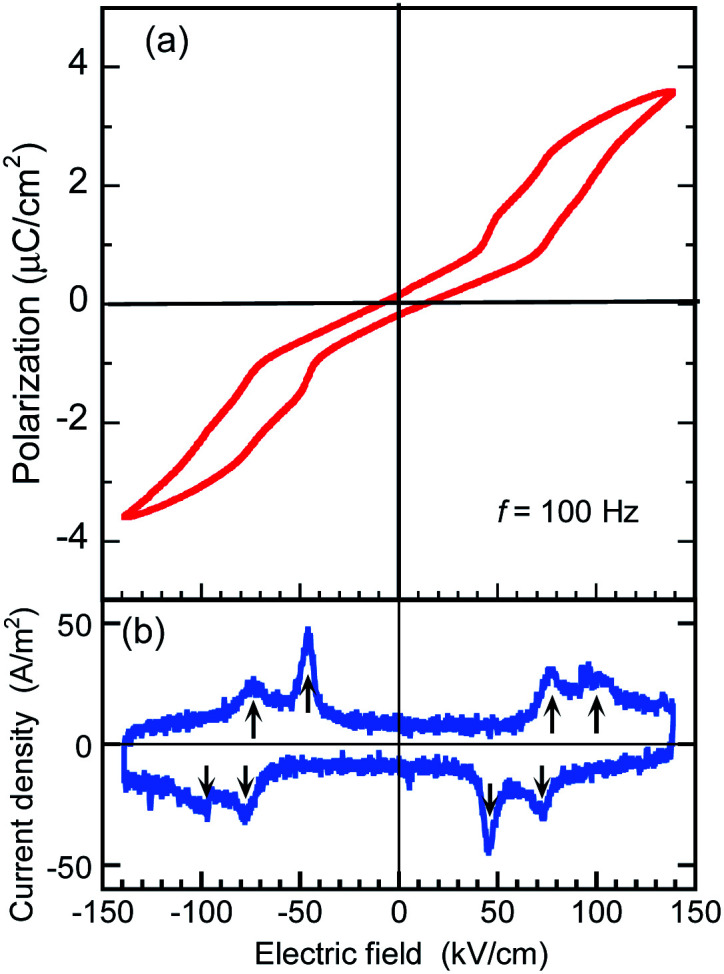
Polarization switching of a sublimed BI2C(s) crystal measured with the applied triangular waveform voltage of *f* = 100 Hz and *E*‖‖〈101〉101‖〈101〉_t_ configuration at room temperature (phase II). (a) *P*–*E* hysteresis loop. (b) Displacement current density indicative of multiple field-induced switching obtained from the corresponding *P*–*E* hysteresis loops.

The BI2C crystals in the phase II were found to reveal a *P*–*E* hysteresis loop characteristic of metaelectric transition with the applied ac electric field configuration *E*‖‖〈101〉101‖〈101〉_t_. Because most of the as-grown crystals are usually twinned by mixing the tetragonal *a* and *b* axes, each monoclinic crystal domain experiences field configuration of either *E*‖‖〈110〉 or 110‖〈110〉 or or *E*‖‖〈011〉 depending on its orientation. The observed 011‖〈011〉 depending on its orientation. The observed depending on its orientation. The observed *P*–*E* hysteresis curves have no spontaneous polarization but double hysteresis loops manifest themselves the metaelectric transition (Fig. 7a), as expected from the structural reassessment above. The quasi-step-like polarization changes of 2.0 μC cm^–2^ emerge at a field amplitude of about 80 kV cm^–1^ and the overall features do not depend on the crystal growth conditions (*i.e.* recrystallization (BI2C(r/EtOH)) and sublimation (BI2C(s))). The reproducible emergence of doublet peaks in displacement current (arrows in Fig. 7b) suggests the successive switching processes.

**Fig. 8:**
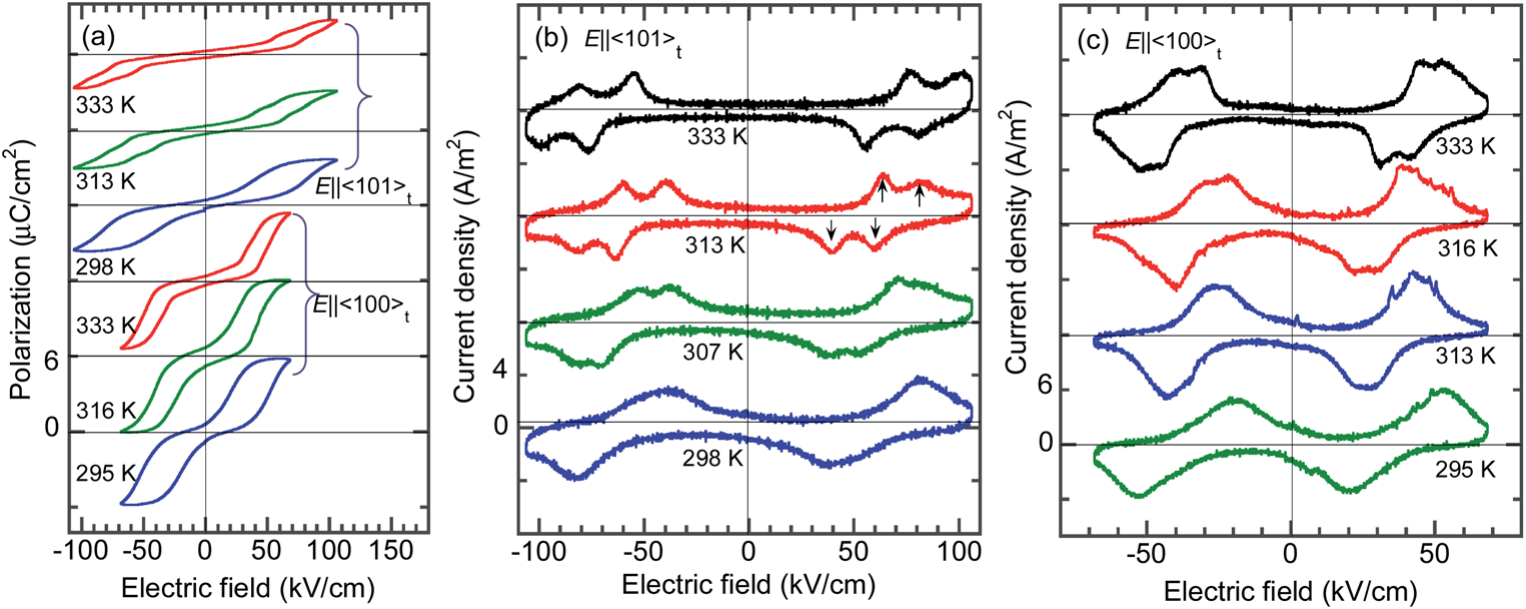
Temperature variation of polarization switching of a deuterated BI2C-*d*_2_(91% D) crystal measured with the applied triangular waveform voltage of *f* = 100 Hz and *E*‖‖〈101〉101‖〈101〉_t_ or *E*‖‖〈100〉100‖〈100〉_t_ configuration. (a) *P*–*E* hysteresis loop. (b) Displacement current density obtained from the corresponding *P*–*E* hysteresis loops. The thermal change in the field-induced switching from a single to a double-step corresponds to the phase III-to-II transition.

The *P*–*E* hysteresis properties of a deuterated BI2C-*d*_2_ crystal have been examined at room and higher temperatures in order to investigate the effect of the phase transition. The triangular waveform voltage was applied with electric field configurations of *E*‖‖〈101〉101‖〈101〉_t_ or *E*‖‖〈100〉100‖〈100〉_t_. The double *P*–*E* hysteresis loops indicative of the metaelectric transition remain in the entire temperature range investigated (Fig. 8a). While the doublet peaks appear in the displacement current in high temperature monoclinic phase II, they are shifted to a lower field and merged into a singlet peak in low temperature tetragonal phase III, reproducibly for both field configurations (Fig. 8b and c). Note that the *E*‖‖〈101〉101‖〈101〉_t_ configuration experiments found more resolved doublet peaks at the higher field than the *E*‖‖〈100〉100‖〈100〉_t_. The latter configuration also resulted in larger polarization changes of ∼6 μC cm^–2^. These anisotropic behaviors can be reasonably explained by the directions of switchable dipole moments, which are parallel to the molecular ribbons extending in the . These anisotropic behaviors can be reasonably explained by the directions of switchable dipole moments, which are parallel to the molecular ribbons extending in the 〈100〉100. These anisotropic behaviors can be reasonably explained by the directions of switchable dipole moments, which are parallel to the molecular ribbons extending in the 〈100〉_t_ directions. The increment of the switching field (36 → 59 kV cm^–1^) and the reduction of polarization (4.1 → 2.5 μC cm^–2^) commonly exhibited a factor of 1.6, which is qualitatively explained by sec *θ* = 1.32 deduced from the inclination angle (*θ* = 40.7°) of the = 40.7°) of the 〈101〉101 = 40.7°) of the 〈101〉_t_ to to 〈100〉100 to 〈100〉_t_ direction.

Attempts to detect the switchable polarization along the [001]_t_ direction failed probably due to the tiny spontaneous polarization as theoretically predicted below.

### Structural mechanism of switching

**Fig. 9:**
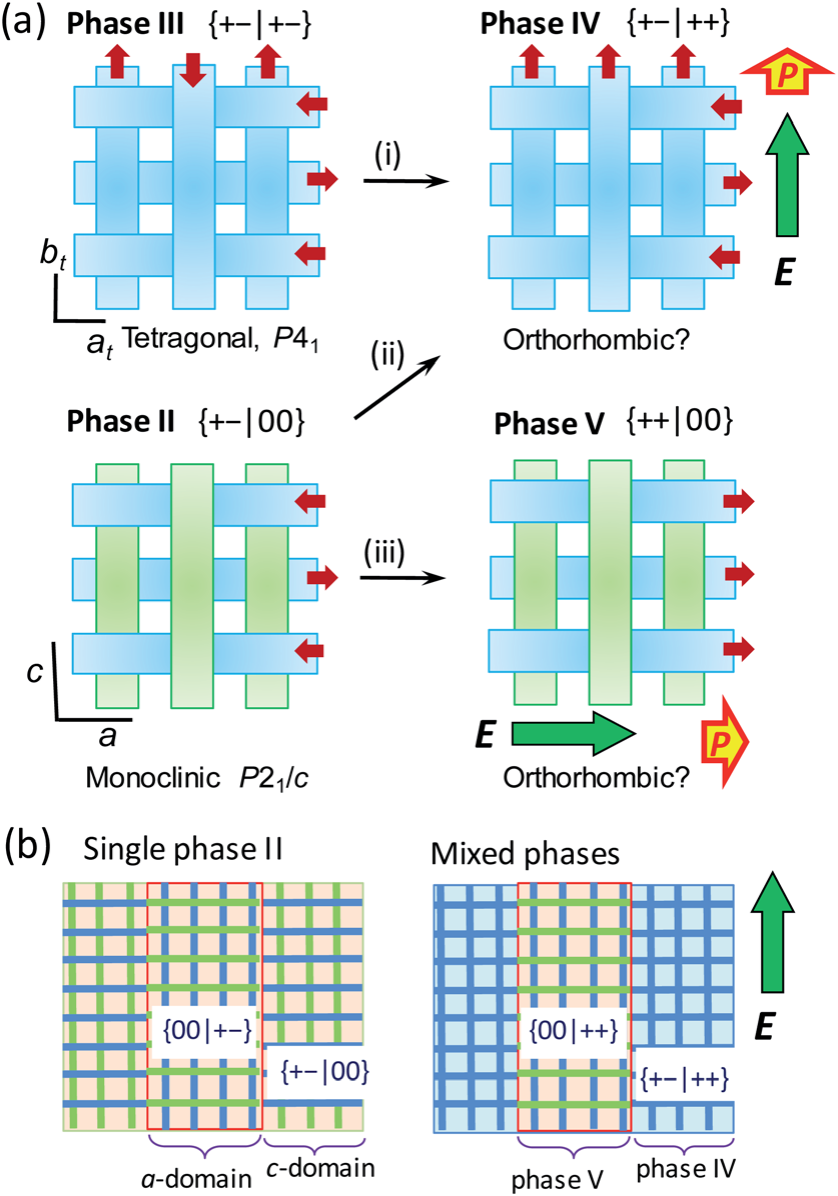
Schematic changes of three-dimensional arrangements of nonpolar and polar ribbons (drawn in green and blue tapes, respectively) induced by an external field *E* applied along the crystallographic *a* or *c* direction (thick arrows). (a) Local structural changes. The low-temperature tetragonal phase III (top, left) exhibiting a single polarization jump through antiferroelectric switching (i). The intermediate monoclinic phase II (bottom, left) exhibiting double polarization jumps due to metaelectric switching (ii) and antiferroelectric switching (iii). (b) Transformation of the multidomain structure of phase II into a phase mixture. The mixture of crystal *a*- and *c*-domains, which refer to those experiencing the field configuration of *E*‖*a* and *E*‖*c*, respectively, are transformed into phase V and IV domains, respectively.

Here we argue the possible structural mechanisms of successive metaelectric transitions in phase II in contrast to the single one in the phase III. Let us recall the switching behaviors of the BI2C-*d*_2_ crystal in the *E*‖‖〈100〉100‖〈100〉_t_ configuration (Fig. 8b). The schematic illustrations in Fig. 9a depict the structural changes in an external field applied in the upper direction of the panel. The tetragonal phase III structure comprises orthogonal arrays of homogeneously polarized ribbons B and the single polarization jump is unambiguously related to the step (i), which flips only the ribbons' dipoles of antiparallel orientation to the field. The field-induced polar form has a {+–|++} type dipole arrangement, and is a new crystal phase (designated as phase IV hereafter), the symmetry of which is plausibly reduced to orthorhombic.

The validity of this model was also checked by the agreement between the experimental results and quantum simulations of electric polarization based on Berry phase theory. In the phase II structure, each ribbon A is located on the inversion symmetry, and then has no net dipole moment. Therefore, polarization was evaluated only with the ribbons B in both phase II and III structures. For simplicity, a polar periodic lattice has been built by leaving only the ribbon B of hydrogen atoms displaced in the +*x* direction. The simulations on phase II and III structures resulted in almost the same polarization (Fig. S10†), which has only the longitudinal (*x*-direction) components. The field-induced polarization requires the doubling of this polarization, because two ribbons B penetrate each unit cell. Thus the obtained theoretical polarization of 4.5–4.6 μC cm^–2^ explains well the observed polarization jump of 4.1 μC cm^–2^, supporting the proposed switching model.

In the phase II structure with {*μ*_1*x*_*μ*_2*x*_|*μ*_3*y*_*μ*_4*y*_} = {+–|00} dipole arrangement, the reduction from tetragonal to monoclinic symmetry leads to a multi-domain state so that each domain experiences the field configuration of *E*‖*a* or *E*‖*c* (Fig. 9b). For the *c*-domains defined by the field *E*‖*c*, the ribbon A being parallel to the field can be transformed into the ribbon B, and thus the emerged polar {+–|++} state is just the same as that of phase IV (step (ii)). On the other hand, the crystal *a*-domains are expected to flip the ribbon B's dipoles antiparallel to the field (step (iii)). The field-induced {++|00} state retains the 1 : 1 mixture of the conformers A and B, and then should be distinguished as a new phase V from IV. Because the two different field-induced phases IV and V depend solely on the applied field configurations, the phase II crystals in the multi-domain state permit the phase separation into phases IV and V. The metaelectric multiphase transitions are naturally explained by the difference in the critical fields between the two phase transitions (steps (ii) *vs.* (iii)).

**Fig. 10:**
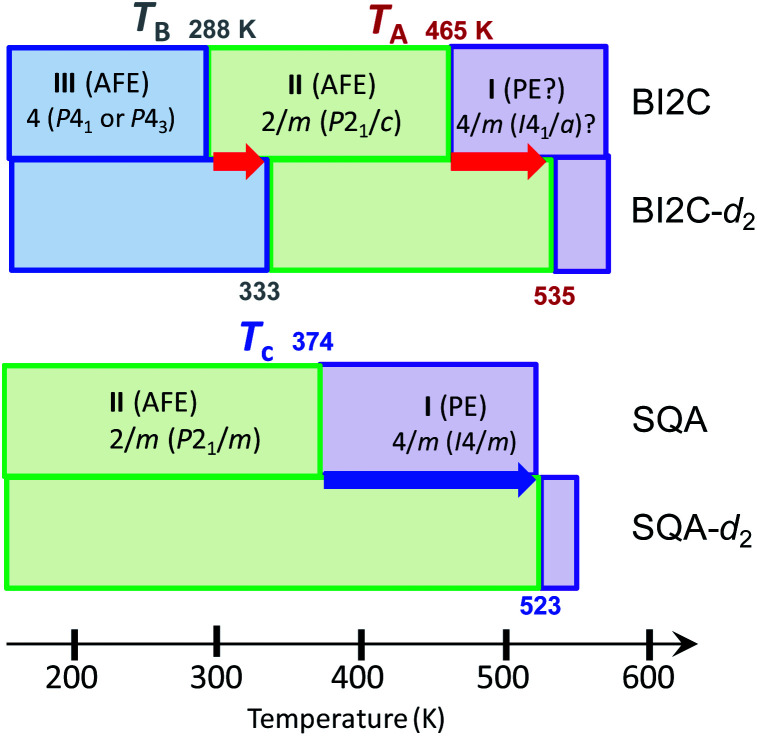
Phase transition sequences of BI2C and SQA. The phase transition temperatures given in kelvin are defined at the midpoints of the thermal hysteresis in the DSC thermogram for the BI2C(s) and BI2C-*d*_2_(91% D) specimens.

It should be noted that the BI2C and SQA crystals^44^–^46^ happen to be similar to each other in the two-dimensional nature of metaelectric properties and the potentially ferroelastic nature with pseudo-tetragonal monoclinic lattice symmetry (point group: 4/*m* → 2/*m*). Furthermore, the multiple field-induced ferroelectric (FE-α and FE-β) phases have been theoretically predicted as well on the SQA crystal although only FE-α is experimentally induced.^28^,^29^ One of the differences is the phase diagram, which of BI2C is more complicated than that of SQA (Fig. 10). We can also see critical differences in molecular symmetry and switching mechanisms between the two dielectrics. According to the “sublattice” model proposed by Kittel for antiferroelectrics,^47^ the switching process can be described by rotating or flipping the polarization of the sublattice. The SQA crystal has a well-defined sublattice, that is, a hydrogen-bonded sheet comprising only molecules of single polarity. In contrast, the ribbons of BI2C cannot represent the sublattice, because their own polarization is also variable between the configurations A and B. The true sublattice, which is constituted by a half (a single benzimidazole) moiety rather than a whole molecular unit, permits only 180°-inversion (not the 90°-rotation) of its polarization unlike the SQA sheet. The model should be much more complicated, considering both the inter- and intra-ribbon dipole–dipole interactions and their interplay.

## Conclusions

A variety of phase change phenomena have been successfully demonstrated in BI2C crystals by virtue of their metadielectric molecular properties. Here, two imidazolyl subunits accommodating each switchable dipole are covalently linked so as to make their total polarization adjustable depending on the relative dipole orientations. Consequently, like a magnetic system of spin *S* = 1, the hydrogen-bonded ribbons can adopt three different states in terms of their dipole moments *μ*: “0” state for the nonpolar ribbons A and “+” or “–” state for the dipolar ones B. Two distinct switching processes are found to describe both temperature- and field-induced structural changes consistently. One is conventional “antiferroelectric” switching with forced alignment of antiparallel dipoles. This mechanism is involved in the field-induced switching from both the phases II and III. The other is “metadielectric” molecular transformation turning on and off the polarization of each ribbon. The latter process is temperature-induced at *T*_II/III_ as well as in either of the metaelectric multiphase transitions.

The other intriguing feature of BI2C crystals arises from ferroelastic-like twinning into a multi-domain state. In the applied electric field, each domain would be transformed into either of the new phases IV and V according to the field configurations. The metaelectric multiphase transitions can be ascribed to the phase transitions II//IV and II/V occurring at different critical fields, which interchange the multi-domain state of single phase II and inhomogeneously mixed state of binary phases IV and V. At first glance, the BI2C crystal is similar to SQA crystals in the two-dimensional nature of antiferroelectricity on the pseudotetragonal lattice and the symmetry reduction from the prototype form. However, its microscopic switching mechanism as well as the sublattice model are quite distinct from each other.

The present work has demonstrated that metadielectric molecular properties actually open the door to rich varieties of electric, thermal, and structural phases, properties, and functionalities of organic dielectrics. The design principle employed herein is quite simple, utilizing only two polar switchable subunits per molecule. For future prospects, there should be many other metadielectric molecules available because the subunits can be modified in diverse ways. It is also expected that further exotic materials should be materialized by increasing the number of subunits.

## Conflicts of interest

There are no conflicts to declare.

## Supplementary Material

Supplementary information

Crystal structure data
