# Supplementary material for: Metaelectric multiphase transitions in a highly polarizable molecular crystal
Source: Chem Sci. 2020 May 13;11(24):6183–92. doi: 10.1039/d0sc01687j (PMC7441576; doi:10.1039/d0sc01687j)
Supplement: Supplementary file 1 [file SC-011-D0SC01687J-s001.pdf]

Electronic Supplementary Material (ESI) for Chemical Science.
This journal is © The Royal Society of Chemistry 2020

Metaelectric multiphase transitions in a highly polarizable molecular crystal

Sachio Horiuchi,[*,†] Shoji Ishibashi,[‡] Rie Haruki,[||] Reiji Kumai,[||] Satoshi Inada,[§] and Shigenobu Aoyagi,[§]

[†]Research Institute for Advanced Electronics and Photonics (RIAEP), National Institute of Advanced Industrial Science and Technology (AIST), Tsukuba 305-8565, Japan

[‡]Research Center for Computational Design of Advanced Functional Materials (CD-FMat), National Institute of Advanced Industrial Science and Technology (AIST), Tsukuba 305-8568, Japan

[||]Condensed Matter Research Center (CMRC) and Photon Factory, Institute of Materials Structure Science, High Energy Accelerator Research Organization (KEK), Tsukuba 305-0801, Japan

[§]Research & Development Center, Ouchi Shinko Chemical Industrial Co., Ltd., Sukagawa 962-0806, Japan

Electronic Supplementary Information

Tables of contents **Pages**

1. Photograph of crystals S2
2. Solvated crystal form S2
3. Thermal properties S3
4. Structural properties S4
5. Electric properties S9

1. Photographs of crystals

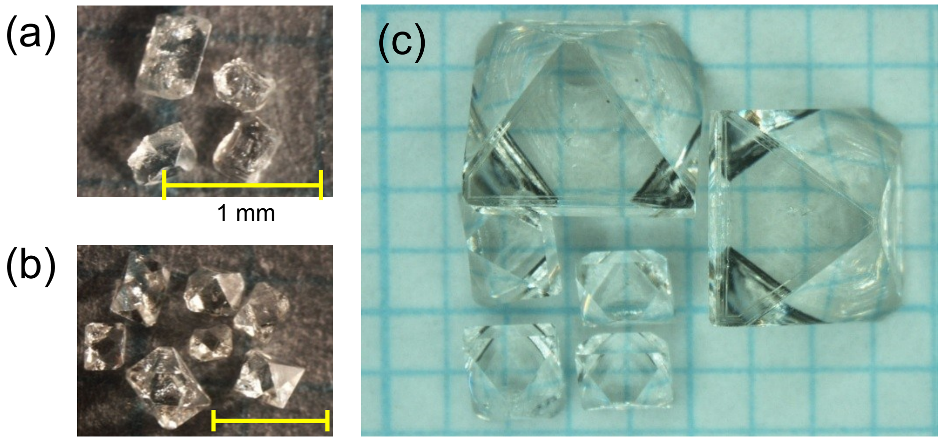

Figure S1. Photographs of the single crystals. (a) Sublimed specimen and (b) recrystallized BI2C crystals. (c) Recrystallized BI2C-d_2 crystals. The scale bars equal to 1 mm in length.

2. Structural properties

Crystal Structure of Solvated BI2C·½MeOH.

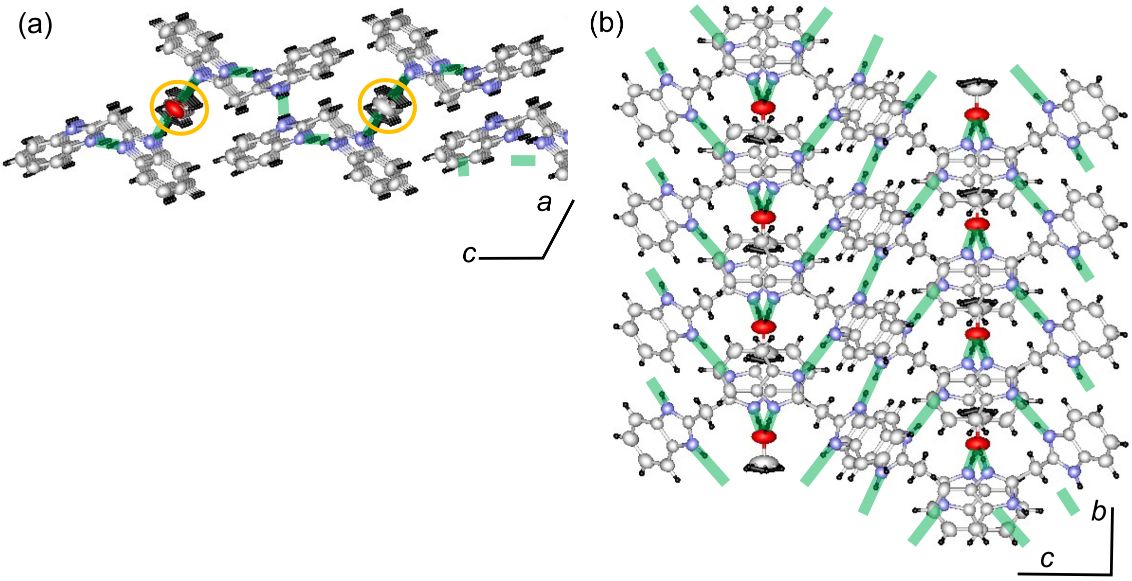

Figure S2. Layered architectures constructed by the intermolecular hydrogen bonds (green thick lines) with solvated methanol in BI2C·½MeOH. (a) Solvent channels (orange circles) parallel to the *b*-direction. (b) Hydrogen-bonded two-dimensional network parallel to the *bc* plane.

The BI2C·½MeOH crystallizes into a layered architecture. The solvent molecules are incorporated in the channels, which are extended parallel to the crystal *b*-direction (orange circles in Figure S2a). This channel structure can explain the observed easy loss of the methanol molecules when the crystal

is exposed to wet air. Due to the 2-fold rotation symmetry on the channel, each methanol molecule therein adopts 2-fold orientational disorder of OH hydrogen atom, so that its short hydrogen bond (N···O distance of 2.775 Å) with two adjacent benzimidazolyl moieties represents the averaged configurations of OH···N and NH···O. The solvent channels are linked with each other through the nearly coplanar four benzimidazolyl moieties with short intermolecular NH···N hydrogen bonds (N···N distance of 2.884 and 3.076 Å) (Figure S2b). The cooperativity of the hydrogen-bonded sequence is a likely reason why both the methanol molecule and benzimidazolyl moiety are involved in the orientational disorder of hydrogen atoms.

3. Thermal properties

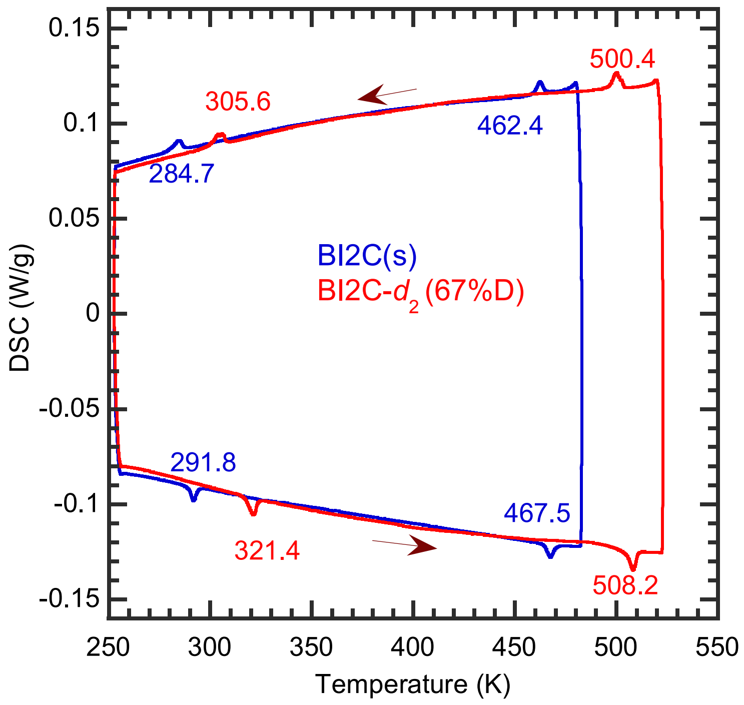

Figure S3. Differential scanning calorimetry (DSC) thermographs of BI2C: sublimed (s) and deuterated (67%D) crystalline solids. Arrows indicate the directions of temperature changes at a rate of 5 K min^{-1}.

4. Structural properties

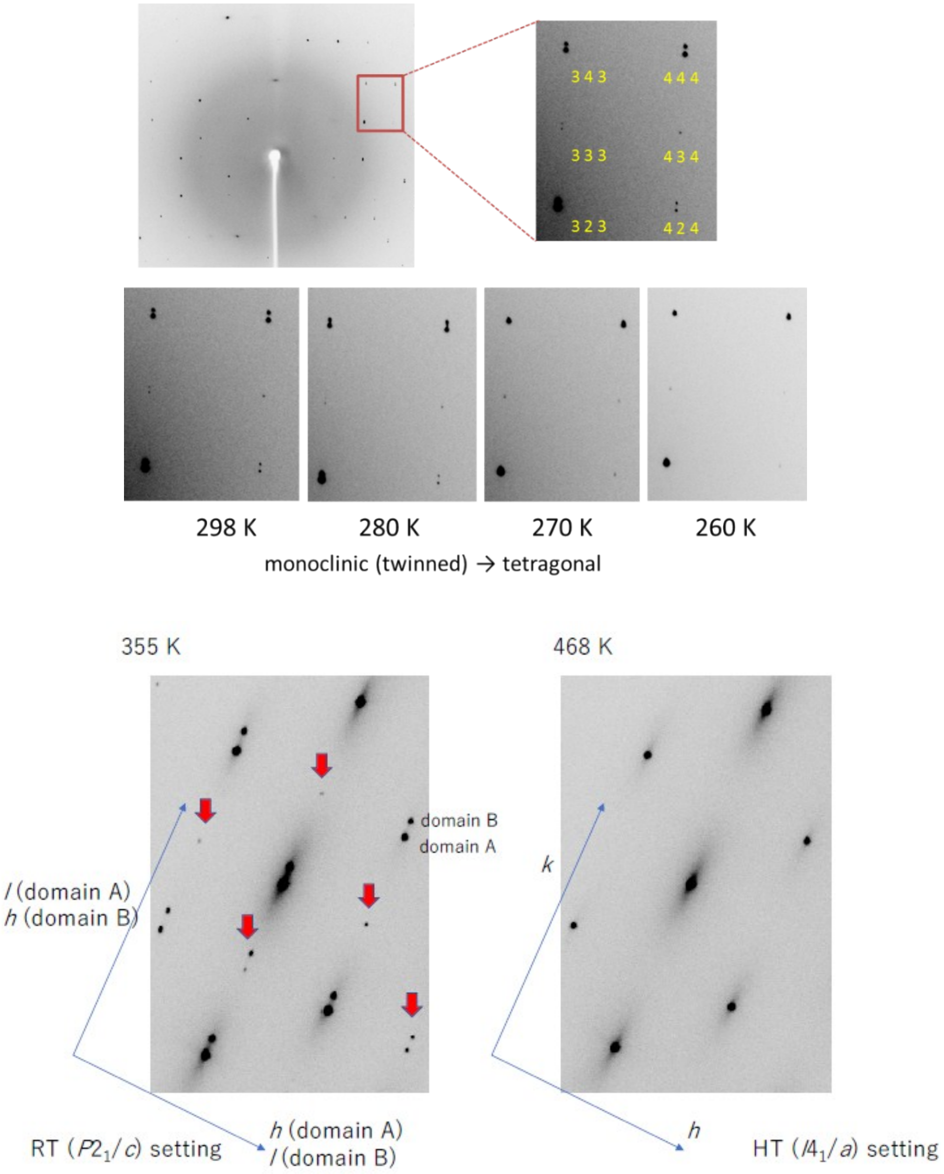

Figure S4. X-ray diffraction images of a sublimed BI2C(s) crystal at various temperatures showing that the phase transition to tetragonal phase III (upper) and phase I (bottom) vanishes the multidomain structure.

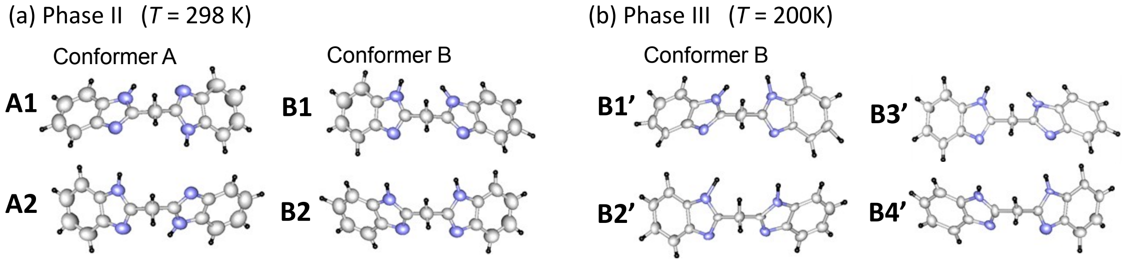

Figure S5. The crystallographically independent BI2C molecular structures viewed along the 2-fold rotation axis of the bridging C-CH_2-C tetrahedron in the crystal structure of (a) phase II (T = 295 K) and (b) phase III (T = 200 K).

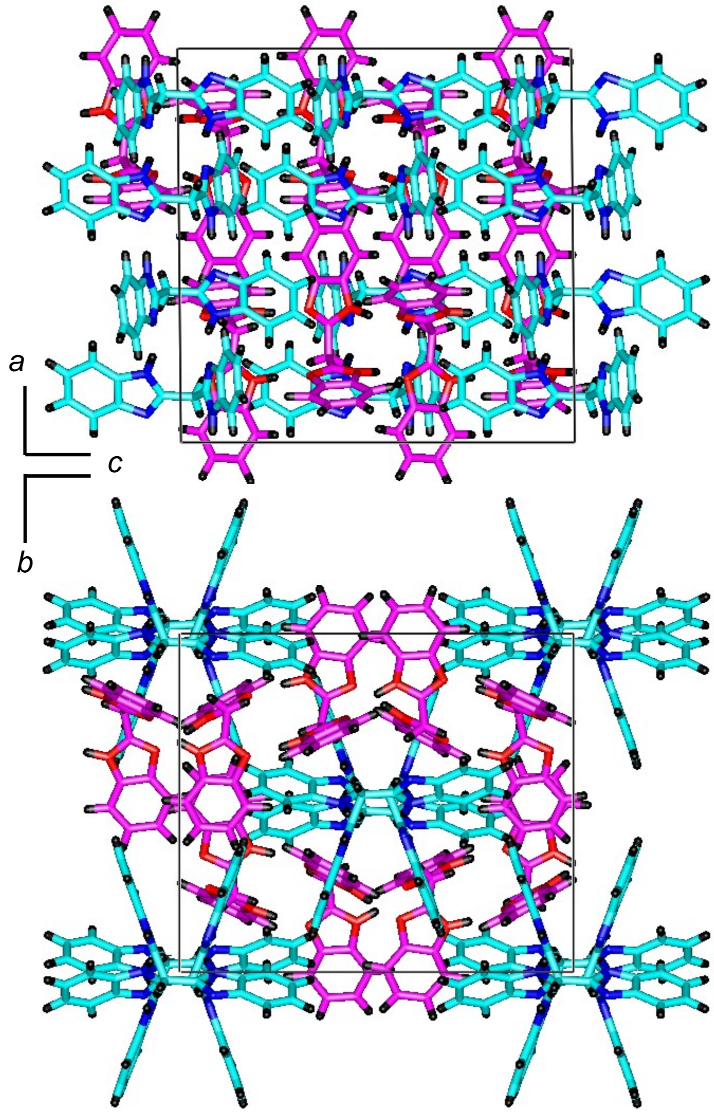

Figure S6. Crystal structure of BI2C viewed along the monoclinic *b* (top) and *a*-axis (bottom) determined at room temperature using the synchrotron x-ray source. Conformers A and B are distinguished by the blue and red colors, respectively.

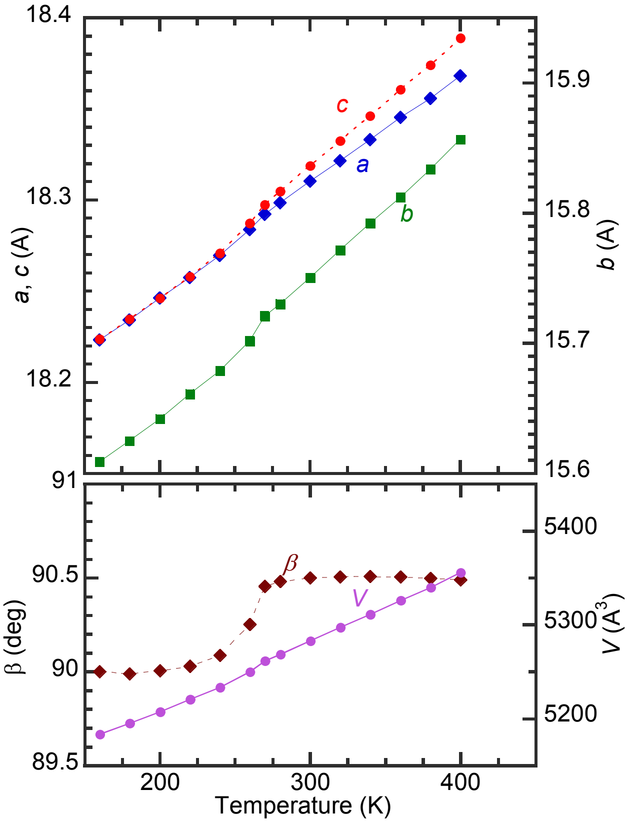

Figure S7. Temperature dependence of unit cell parameters of a recrystallized BI2C(r/EtOH) crystal.

Table S1. Crystallographic data collection and structural refinement information for BI2C and its solvate.

	BI2C(r/EtOH)		BI2C(s)	BI2C-d_2(91%D)	BI2C·½MeOH
Temperature, K	293	160	295	295	293
chemical formula	$C_{15}H_{12}N_4$			$C_{15}D_2H_{12}N_4$	$C_{31}H_{28}N_8O_1$
crystal system	monoclinic	tetragonal	monoclinic	tetragonal	monoclinic
space group	$P2_1/c$ (#14)	$P4_1$ (#76)	$P2_1/c$ (#14)	$P4_3$ (#78)	$C2/c$ (#15)
a, Å	18.3346(7)	18.2293(3)	18.3296(17)	18.3126(4)	22.088(3)
b, Å	15.7546(3)	18.2293(3)	15.7592(14)	18.3126(4)	5.5860(7)
c, Å	18.3241(3)	15.6088(7)	18.3256(16)	15.7252(5)	24.017(3)
α, deg	90	90	90	90	90
β, deg	90.531(1)	90	90.610(3)	90	115.257(3)
γ, deg	90	90	90	90	90
V, Å^3	5292.8(2)	5186.9(3)	5293.2(8)	5273.5(2)	2680.0(6)
Z (Z')	16 (4)	16 (4)	16 (4)	16 (4)	8 (1)
D_{calc}, g cm^{-3}	1.246	1.272	1.246	1.251	1.310
Dimensions, mm	0.10×0.10×0.05	0.10×0.10×0.05	0.38×0.25×0.23	0.38×0.34×0.33	0.35×0.21×0.07
Radiation [a]	Synchrotron, λ = 1.00 Å		MoKα (C)	MoKα (P)	MoKα (C)
$2\theta_{max}$, deg	92	130	55	55	55
R_{int}	0.0160	0.0232	0.034	0.0105	0.024
reflcn used ($2\sigma(I)<I$)	9236	18269	12000	11425	3047
no. of variables	718	685	718	685	199
R, wR	0.0400, 0.1173	0.0571,0.1749	0.067, 0.1908	0.047, 0.1527	0.041, 0.102
GOF	0.909	1.067	1.051	1.014	1.025
$d_{N\cdots N}^{(min)}$, $d_{N\cdots N}^{(max)}$; $d_{N\cdots N}^{(av)}$, Å	2.771, 2.850; 2.804	2.759, 2.847; 2.784	2.773, 2.852; 2.805	2.775, 2.860; 2.800	

[a] (C), CCD area detector; (P), hybrid pixel detector.

Table S2. Torsion angles of two imidazole rings about each methylene C-CH_2-C bridge for BI2C and its deuterated crystals. The italic and roman characters represent those of the conformers A and B, respectively.

Mol #	Ring #	BI2C(s)<br>$\varphi_i^{(1H)}$, $\varphi_i^{(2H)}$, deg	BI2C-d_2(91%D)<br>$\varphi_i^{(1D)}$, $\varphi_i^{(2D)}$, deg
I	1 (Φ_1)	-134.5(2)<br>C32 C31 C39 N12<br>49.8(3)<br>C32 C31 C39 N11*	-132.2(3)<br>C32 C31 C39 N11*<br>50.8(5)<br>C32 C31 C39 N12
	2 (Φ_2)	95.2(2)<br>C39 C31 C32 N9*<br>-83.2(3)<br>C39 C31 C32 N10	100.2(3)<br>C39 C31 C32 N9*<br>-78.7(4)<br>C39 C31 C32 N10
II	3 (Φ_3)	-80.2(3)<br>C47 C46 C54 N16<br>98.3(2)<br>C47 C46 C54 N15*	-85.8(4)<br>C47 C46 C54 N15*<br>93.9(4)<br>C47 C46 C54 N16
	4 (Φ_4)	49.7(3)<br>C54 C46 C47 N13*<br>-133.3(2)<br>C54 C46 C47 N14	57.3(4)<br>C54 C46 C47 N13*<br>-126.9(3)<br>C54 C46 C47 N14
III	5 (Φ_5)	130.28(19)<br>C2 C1 C9 N3*<br>-51.8(3)<br>C2 C1 C9 N4	129.6(3)<br>C2 C1 C9 N3*<br>-53.2(4)<br>C2 C1 C9 N4
	6 (Φ_6)	-102.6(2)<br>C9 C1 C2 N1*<br>76.4(3)<br>C9 C1 C2 N2	-101.0(4)<br>C9 C1 C2 N1*<br>78.4(4)<br>C9 C1 C2 N2
IV	7 (Φ_7)	85.1(3)<br>C17 C16 C24 N7*<br>-94.6(3)<br>C17 C16 C24 N8	86.4(4)<br>C17 C16 C24 N7*<br>-91.7(4)<br>C17 C16 C24 N8
	8 (Φ_8)	-57.6(3)<br>C24 C16 C17 N5*<br>126.0(2)<br>C24 C16 C17 N6	-56.6(4)<br>C24 C16 C17 N5*<br>126.3(3)<br>C24 C16 C17 N6

* The NH nitrogen atoms of imidazole rings are marked by asterisks.

See the illustration below for definition of the CCCN torsion angles.

The dihedral angles Φ_i taken as positive 0-180° are defined by averaging torsion angles as

$$\Phi_i = (\varphi_i^{(1H)} + \varphi_i^{(2H)} + 180)/2.$$

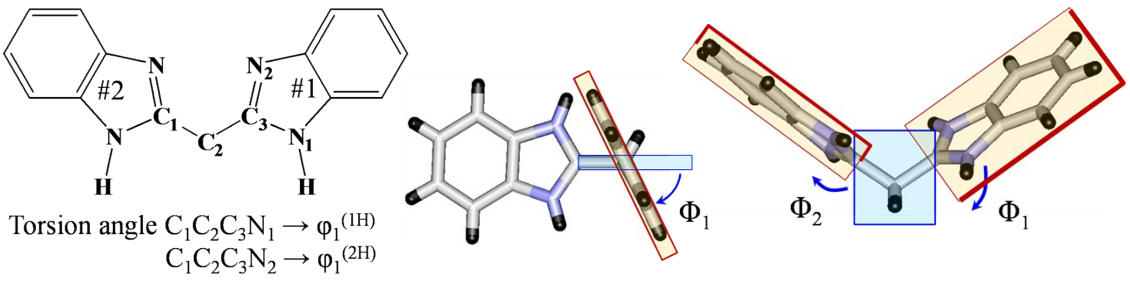

5. Electric properties

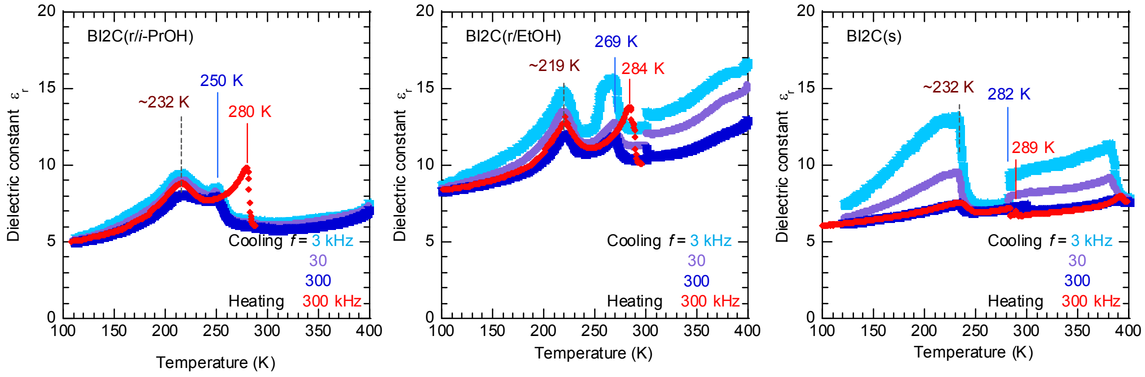

Figure S8. Temperature-dependent relative permittivity ε_r measured with an ac field of various frequencies of $E\|[101]_t$ configuration; BI2C(r/*i*-PrOH) and BI2C(r/EtOH) specimens recrystallized from iso-propylalcohol and ethanol, respectively (left and middle panels) and sublimed BI2C(s) specimen (right panel). The temperatures of phase transition in the cooling/heating runs and those of the broad permittivity maxima are given in blue/red and brown, respectively.

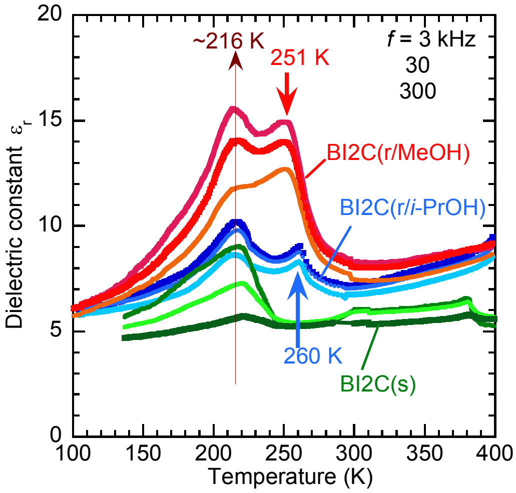

Figure S9. Temperature-dependent relative permittivity ε_r measured in the cooling runs with an ac field of various frequencies of $E\|[101]_t$ configuration; BI2C(r/MeOH) and BI2C(r/*i*-PrOH) specimens recrystallized from methanol and iso-propylalcohol, respectively, and sublimed BI2C(s) specimen (right).

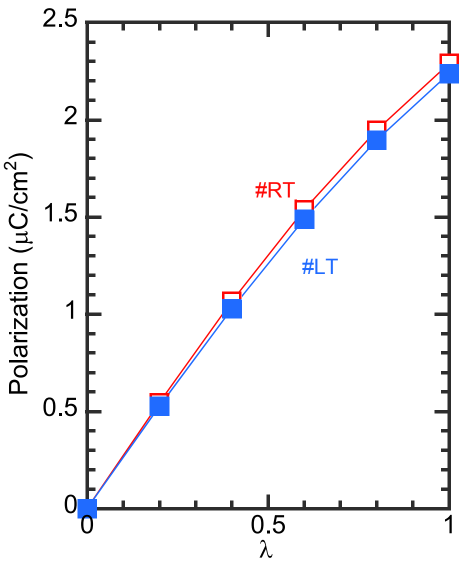

Figure S10. Variation of the theoretical polarizations of a ribbon B as a function of degree of polar distortion λ changing from the centrosymmetric reference (paraelectric, λ = 0) to the fully polarized (ferroelectric, λ = 1) configurations. Note that the polarization has only longitudinal direction component along the ribbon. Ribbon B is selected from phase III (#LT, T = 200 K) or phase II (#RT, T = 295 K) structure.
